# Alcohol consumption/dependence and resilience in older adults with high blood pressure^1^

**DOI:** 10.1590/1518-8345.2466.3024

**Published:** 2018-08-09

**Authors:** Aline Alves dos Santos Dullius, Silvana Maria Coelho Leite Fava, Patrícia Mônica Ribeiro, Fábio de Souza Terra

**Affiliations:** 2 MSc, RN, Secretaria Municipal de Saúde, Prefeitura Municipal de Machado, Machado, MG, Brazil.; 3 PhD, Associate Professor, Escola de Enfermagem, Universidade Federal de Alfenas, Alfenas, MG, Brazil.; 4 PhD, Adjunct Professor, Escola de Enfermagem, Universidade Federal de Alfenas, Alfenas, MG, Brazil.

**Keywords:** Aged, Hypertension, Alcoholism, Psychological Resilience, Family Health Strategy, Nursing

## Abstract

**Objective:**

to evaluate alcohol consumption/dependence and resilience in older adults with high blood pressure and to analyze the factors associated with these variables.

**Method:**

a descriptive, cross-sectional, quantitative study developed with 300 older adult patients with high blood pressure from Family Health Strategy units in a municipality of Minas Gerais, Brazil. A semi-structured questionnaire called the *Alcohol Use Disorder Identification Test* and the Resilience Scale were used. Data were analyzed using the Pearson’s chi-square test, Fisher’s exact test, Cronbach’s alpha, odds ratio and logistic regression.

**Results:**

89.3% of the interviewees were low-risk for consuming alcoholic beverages. The variables gender, age, smoking and disease duration were significantly associated with alcohol consumption/dependence. 36.7% of the people presented a low resilience. The variables family and individual monthly income, education level, physical activity and leisure had an association with resilience. No statistically significant association was observed between alcohol consumption/dependence and resilience.

**Conclusion:**

alcohol consumption and resilience can interfere with the physical and mental health of older adults with high blood pressure.

## Introduction

The Brazilian population has undergone transformation processes characterized by significant changes in its demographic regime. The levels and patterns of vital events, fecundity and mortality experienced in all regions of the country have been changing rapidly in recent decades, implying in challenges and opportunities for society^(^¹^)^. Along with this phenomenon, the numbers of non-transmissible chronic diseases such as high blood pressure (HBP)^2^ and alcohol consumption by the older adult population have also increased^3^ ^-^ ^5^.

North American data from 2015 established a relationship between HBP and acute myocardial infarction in 69% of cases, as well as in 77% of cases of hemorrhagic stroke, 75% of heart failure, and was also responsible for 45% of cardiac deaths and 51% of deaths due to hemorrhagic stroke. In Brazil, HBP affects approximately 36 million adult individuals and more than 60% of the older adult population, with high impact on loss of labor productivity and family income^6^ ^-^ ^7^.

Therefore, we can highlight the need for nurses to seek early identification of diseases and complications that may negatively impact adherence to HBP treatment, such as the presence of comorbidities^8^.

In this context, it should be noted that alcohol use among older adults is simultaneously increasing with the growth of this population. Thus, alcoholism can be responsible for serious social issues, representing a little studied and diagnosed public health problem in certain populations. Studies related to alcohol consumption are very concentrated among young people/adults, demanding new perspectives for this problem with the adoption of appropriate identification and treatment techniques among the older adult population^9^.

In many situations it is necessary for the individual to use strategies to cope with events in their life, including the onset of illness or changes to their lifestyle. Thus, the personality trait known as resilience is understood as the healthy and positive development of the individual influenced by social and intrapsychic processes, even when experiencing unfavorable experiences. In this sense, resilience involves interaction between adverse life events and internal and external protective factors of each individual^10^. 

A study conducted with 2,024 adults in Atlanta, USA, assessed the relationship between alcohol and resilience, and found that individuals with low resilience presented higher rates of problems related to alcohol consumption/dependence, tobacco and other drugs^11^.

For older adults, resilience also appears as a protective factor for abuse of harmful substances such as alcohol consumption. In contrast, it has been identified as one of the factors which contribute to successful aging, which refers to the way the elderly achieve and maintain their sense of well-being despite the natural difficulties that appear with aging. At this stage of life, resilience is related to greater social engagement, greater optimism, and functional independence^8^.

Thus, if resilience in older adults with HBP is strengthened, they are expected to seek periodic follow-up for their chronic condition and to correctly follow drug and non-drug treatment, with the abandonment of alcoholism among the treatments, in order to achieve successful aging^9^. 

Despite identifying the importance of this subject, studies that address resilience and alcohol consumption/dependence in older adults with HBP are still scarce, which justifies the search for knowledge in this area in order to elucidate the prevalence and influence of these factors at this stage of life, and thus contribute to improving nursing care to this portion of the population. Based on this information, nurses can develop actions to promote resilience such as stimulus to self-esteem, as well as contribute to preventing, reducing or abandoning the consumption of alcoholic beverages. All these actions will favor controlling blood pressure levels in the older adult population and contribute to their social and individual well-being, as well as improving the quality of life of this population.

Thus, the objective of the present study was to evaluate alcohol consumption/dependence and the resilience of older adults with high blood pressure attended by Family Health Teams of a municipality in the South of Minas Gerais, and to analyze the factors associated with these variables.

## Method

A descriptive, cross-sectional, quantitative study developed in five urban Family Health Strategy units (*Estratégia Saúde da Família* - *ESF*) in a municipality in the South of Minas Gerais, in the period between October of 2015 and February of 2016. 

The study population consisted of 1092 older adults who only presented HBP as a chronic disease. The following inclusion criteria were adopted: being 60 years old or more; belonging to a population assigned to one of the five urban Family Health Teams of this municipality; presenting high blood pressure; and not having any another chronic diseases. A proportional stratified random sample for each of the five *ESF*s was calculated based on these criteria and the total population, thus totaling a sample of 300 people. The sample size was calculated using 95% confidence and 5% error, and the BioEstat 5.3 program was used for the random selection of the older adults who composed this sample.

To perform the data collection, the nurses of the *ESF* under study were asked to list all the older adults with HBP who are part of the coverage area of each team. The people who composed the sample and who were invited to participate in the present study were then randomly drawn based on this list and according to the sample size calculation. After the draw, the researcher subsequently communicated with the selected individuals to schedule the best time to apply the instruments. 

It should be noted that the interviews for data collection were carried out at the interviewee’s home due to the fact that the study population consisted of older adults who could present visual or reading difficulties or difficulties in filling out the instruments.

A semi-structured questionnaire with 18 questions developed by the researchers and aimed at evaluating sociodemographic data, life habits and chronic illness, work activities and important events in life was used for data collection. This instrument was submitted to a refinement process with five experienced judges in the studied area. It was subsequently submitted to a pilot test with 10 people belonging to an *ESF* of the same municipality. It should be noted that the individuals who participated in this process were not included in the study sample. 

The *Alcohol Use Disorder Identification Test* (AUDIT) was used to assess alcohol consumption/dependence, which is an instrument originally developed by the World Health Organization (WHO) in the late 1980s. The Portuguese version was translated for the Brazilian context in 1999 and adapted in 2005, and this latest version was used in the present study^12^. The classification based on the sum of the ten answers is: a score between 0 to 7 points as low-risk use; a score between 8 to 15 points as risky use; a score between 16 to 19 points as harmful use; and a score between 20 to 40 points as probable dependence. 

A third instrument called the Resilience Scale, developed in 1993 in English and translated and validated in Portuguese in 2005, was used to evaluate resilience^13^. The instrument has 25 items, positively described with Likert-type responses ranging from 1 (totally disagree) to 7 (totally agree). The scoring on this scale can vary from 25 to 175 points, corresponding to: values greater than 145 indicate moderately high to high resilience; 125 to 145 refer to moderately low to moderate resilience levels; and values equal to or less than 124 points correspond to low resilience^14^.

The data collected by the instruments were inserted by double entry into the Statistical Package for Social Science (SPSS) version 20.0 software in order to avoid transcription errors and for descriptive and inferential statistical analysis. 

To assess the reliability of the AUDIT Scale and the Resilience Scale, Cronbach’s Alpha Coefficient was used to evaluate the internal consistency and whether the data collected correlated to one another. 

In order to facilitate the statistical analysis of the data and the comparisons, some independent variables were regrouped. It should be noted that the variable alcohol consumption/dependence was recoded as two categories for the associations: low risk use x risky use. Likewise, the resilience variable was also recoded as two categories with the same intention: low resilience x moderately low to moderate resilience, and moderately high to high resilience.

In addition, Pearson’s Chi-square test or Fisher’s exact test were used to verify the association between alcohol consumption/dependence and independent variables, the resilience measurement with the independent variables, and also the alcohol consumption/dependence variable and the resilience.

The study adopted a significance level of 5%, meaning all data were statistically significant for P<0.05. 

After these analyzes, the *odds ratio* of the independent variables with alcohol consumption/dependence and resilience was estimated. Next, the logistic regression model of the independent variables with alcohol consumption/dependence and resilience was used. 

The research project was submitted for evaluation and appreciation by the Research Ethics Committee of the Federal University of Alfenas (UNIFAL-MG), and approved under opinion number 1.144.940 (CAAE: 46503115.3.0000.5142). A clear and Informed Consent Form was signed by the study participants, guaranteeing their anonymity and their right to withdraw at any stage of the research, according to Resolution 466/2012 which comprises Research Involving Human Beings.

## Results

The sample mostly consisted of female older adult individuals (61.0%), aged between 60 and 70 years (58.0%), married/with partners (58.7%), who were Catholic as a religious belief (79%), had one to five children (61.0%), had their own housing (87.0%), incomplete primary education (51.3%) and were illiterate (34.7%). The monthly family income corresponded to R$1,701 to R$2,500 (27.0%), and individual monthly income of up to R$880.00 (71.7%). Only 13.3% reported being smokers, and 67.5% of these consumed up to 10 cigarettes per day. Most of the interviewees did not practice any physical activities (56.0%) and reported having some leisure activity (93.7%), highlighting manual activities (28.1%), followed by watching television (27.0%). Among the participants, 49.7% had HBP for 1 to 10 years, 93.0% reported continuously using medication to control this pathology, in which most of the interviewees (48.0%) reported using 2 types of medication per day. The most used type of pharmacological drug to control the disease were diuretics (67.7%), followed by Angiotensin II receptor blockers (49.1%). It is important to highlight that only a small part of the older adult population interviewed had some support for their daily use of medication (10.0%), in which they all reported counting on family members as the people who provide this support.

Regarding treatment, 39.7% of the participants reported not performing any type of non-pharmacological treatment for controlling HBP. The older adults who presented complications associated with the disease corresponded to 22.0% of the interviewees, with heart disease (47.0%) being the most frequently reported among them. Regarding their occupation, the majority were rural workers (41.0%), and their current situation was retired (81.3%). When analyzing the variable “remarkable life events”, it was verified that 73.7% had some remarkable event in the last 12 months, with emphasis on the loss (death) of a loved one (38.5%), followed by family conflicts (19.0%), a personal diagnosis of illness (13.1%), or a personal accomplishment (10.4%).

When assessing the distribution of older adults with HBP according to the classification of the AUDIT scale, it was possible to verify that 89.3% of the interviewees had low-risk consumption of alcoholic beverages, 6.0% had risky consumption and 2.0% had harmful consumption. It should be noted that 2.7% of the older adults presented a probable dependence for alcohol consumption.

Table 1 shows the only independent variables that had a significant association with alcohol consumption/dependence among the older adults interviewed.

**Table 1:** Univariate analysis of the factors associated with alcohol consumption/dependence of older adults with high blood pressure according to the variables: gender, age group, smoking, duration/onset of high blood pressure, and continuous use of medications. Machado, MG, Brazil, 2016

Variables	Low-risk consumption	Risky consumption	P-value	OR*	95% CI^†^
Gender					
Males	89 (76.1%)	28 (23.9%)	<0.001^‡^	1.000	0.024 - 0.209
Females	179 (97.8%)	4 (2.2%)		0.071	
Age group					
60 to 70 years	150 (86.2%)	24 (13.8%)	0.039^§^	1.000	0.184 - 0.977
71 years or older	118 (93.7%)	8 (6.3%)		0.424	
Smoking					
Yes	27 (67.5%)	13 (32.5%)	<0.001^‡^	6.107	2.701 - 13.727
No	241 (92.7%)	19 (7.3%)		1.000	
HBP^||^ Duration/onset					
Up to 20 years	196 (87.1%)	29 (12.9%)	0.020^§^	1.000	0.083 - 0.953
More than 20 years	72 (96.0%)	3 (4.0%)		0.282	
Use of continuous medications					
Yes	252 (90.3%)	27 (9.7%)	0.043^‡^	0.343	0.116 - 1.009
No	16 (76.2%)	5 (23.8%)		1.000	

*OR - *Odds ratio*; †CI - Confidence Interval; ‡Fisher’s Exact Test; §Pearson’s Chi-Square Test; ||HBP - High blood pressure.

According to Table 1, only gender, age group, smoking, duration/onset of HBP and continuous use of medication had a significant association with alcohol consumption/dependence among all the independent variables analyzed (P <0.05). Thus, males and participants aged between 60 and 70 years old were more likely to have risky alcohol consumption/dependence. Older adult smokers were 6 times more likely to present risky alcohol consumption/dependence than non-smokers. Also, those interviewed who developed HBP in the last 20 years and those who did not use continuous medications to control this disease were more likely to have risky alcohol consumption/dependence. 

After analyzing the parameters of all independent variables with alcohol consumption/dependence by the logistic regression model, only the variables gender and smoking presented a statistically significant correlation at p<0.001 and p=0.028, respectively, resulting in an adjusted final model. Thus, the model found that male respondents (OR: 1.000) were more likely to present risky alcohol consumption/dependence than females. Furthermore, smokers (OR: 6.107) were approximately 6 times more likely to have risky alcohol consumption/dependence than non-smokers.

Regarding the distribution of the older adults according to the Resilience Scale classification, 39.7% of respondents presented moderately low to moderate resilience, 36.7% low resilience and 23.6% moderately high to high resilience.

Table 2 presents the only independent variables that had a significant association with resilience among the older adults with HBP.

**Table 2:** Univariate analysis of the factors associated with the resilience of older adults with high blood pressure according to the variables: Monthly family income, individual monthly income, education level, physical activity, leisure activity. Machado, MG, Brazil, 2016

Variables	Low Resilience	M.L.M* a M.H.H^†^ Resilience	P-value	OR^‡^	95% CI^§^
Monthly family income					
Up to R$1,700	67 (45.3%)	81 (54.7%)	0.002^||^	2.097	1.299 - 3.385
More than R$1,701	43 (28.3%)	109 (71.7%)		1.000	
Individual monthly income					
Up to R$880.00	94 (40.2%)	140 (59.8%)	0.018^||^	2.098	1.128 - 3.903
More than R$881.00	16 (24.2%)	50 (75.8%)		1.000	
Education level					
Illiterate	51 (49.0%)	53 (51.0%)	0.001^||^	2.234	1.368 - 3.650
Literate	59 (30.1%)	137 (69.9%)		1.000	
Physical activity					
Does not perform	79 (47.0%)	89 (53.0%)	<0.001^||^	2.892	1.748 - 4.786
Performs physical activity	31 (23.5%)	101 (76.5%)		1.000	
Recreational activity					
Yes	99 (35.2%)	182 (64.8%)	0.047^||^	1.000	0.984 - 6.491
No	11 (57.9%)	8 (42.1%)		2.528	

*M.L.M - moderately low to moderate (resilience); †M.H.H - moderately high to high (resilience); ‡OR - *Odds ratio*; §CI - Confidence Interval; ||Pearson’s Chi-Square Test

According to Table 2, the variables monthly family income, individual monthly income, education level, physical activity and leisure activity presented a significant association with resilience (P<0.05). Thus, the participants who presented monthly family income up to R$1,700 (OR: 2.097), as well as those with individual monthly income of up to R$880.00 (OR: 2.098), and illiterate older adults (OR: 2.234) are about two times more likely of having low resilience. Respondents who did not practice physical activity (OR: 2.892) were nearly three times more likely to have low resilience. And finally, individuals who reported not having a leisure activity (OR: 2.528) were 2.5 times more likely to have lower resilience than those who reported having a leisure activity.

After analyzing the parameters of all the independent variables with resilience according to the logistic regression model, it was observed that only the variables education level, physical activity and significant life event had a statistical significance, respectively, p=0.020, P<0.001 and p=0.044, resulting in an adjusted final model. Thus, the model found that people without formal education (illiterate) were approximately 2 times more likely to present low resilience than those with literacy. In addition, respondents who did not perform physical activity were 3 times more likely to have lower resilience than those who did. And finally, the subjects who experienced some significant event in their life were approximately 2 times less likely to present low resilience than those who did not experience any of these events.

Table 3 shows the analysis of the association between alcohol consumption/dependence with resilience.

**Table 3:** Univariate analysis of alcohol consumption/dependence with resilience in older adults with high blood pressure. Machado, MG, Brazil, 2016

Variables	Low Resilience	M.L.M* a M.H.H^†^ Resilience	P-value	OR^‡^	95%CI^§^
Alcohol consumption/ dependence					
Low-risk consumption	95 (35.4%)	173 (64.6%)	0.205^||^	0.622	0.298 - 1.302
Risky consumption	15 (46.9%)	17 (53.1%)		1.000	

*M.L.M - moderately low to moderate (resilience); †M.H.H - moderately high to high (resilience); ‡OR - *Odds ratio*; §CI - Confidence Interval; ||Pearson’s Chi-Square Test

When assessing the association between the variables alcohol consumption/dependence and resilience among the older adults with HBP, no significant association was observed between these two variables (p=0.205). However, it is possible to identify that an expressive percentage of the evaluated subjects had risky consumption of alcoholic beverages and presented low resilience (46.9%).

## Discussion

According to the data obtained in the present study, it was identified that the majority of respondents were females, aged between 60 and 70 years, married/with companions, had Catholicism as a religious belief, with one to five children, lived in their own house, with incomplete primary education and were illiterate. Similar data can be observed in a study conducted in Maranhão with 60 older adults that consisted of 65% of women, 63.3% were aged between 60 and 69 years, 43.3% had incomplete secondary education, and 36.7% of the sample were illiterate^15^. Another study carried out in the Legal Amazon dealt with the epidemiological characteristics of HBP and the factors associated with this pathology in the older adult population, finding similar data to that found by the present investigation; among the 273 older adults analyzed, 54.9% reported between one and four years of education and 34.5% were illiterate^16^. Other authors developed a study with 1047 older adults with HBP in Madrid, Spain, which identified that 36% used angiotensin II receptor blockers, 23.9% ACE inhibitors and 20% used diuretics ^(^ ^17^. 

In the present study it was verified that the variables gender, age group, smoking, duration/onset of HBP and continuous use of medications showed a significant association with the variable alcohol consumption/dependence. 

The literature points out that males are strongly associated with an increase in alcohol consumption in comparison to females. The pattern of alcohol consumption in older adults and the moral and social values associated with this habit can even be determined in youth, lasting for life. In addition, there is strong social pressure for men to start drinking alcoholic beverages when they are younger. In contrast, older women may have experienced an adolescence where this habit was not valued among the female gender, which may have influenced the low alcohol consumption among these women after the aging process^18^.

Regarding the age group, it should be mentioned that there is a decrease in alcohol consumption in old age, and the greater the age, the lower the frequency of its consumption. This is due to some factors such as premature death of people who have used alcohol throughout their lives, moderation or discontinuation in consuming the substance due to increased sensitivity to the effects of alcohol or aspects that influence older adults not reporting their alcohol consumption, and/or decreased investigation by the health team^19^.

Similarly to alcohol consumption, smoking can harm older adult’s health, resulting in various social and economic problems in the country. The literature establishes an association between alcohol consumption and tobacco^20^. 

Another research further revealed that there are a growing number of older adults who have comorbidities such as HBP and diabetes mellitus, and take daily medications to control these diseases, but who (also) consume alcoholic beverages as a coping strategy for their health condition^21^.

In the present study, an association between alcohol consumption/dependence and the fact that some subjects presented a HBP diagnosis for over 20 years was also observed. This finding possibly occurred because people who had a more recent HBP diagnosis were found to be in the initial age group bracket, since consumption of alcoholic beverages decreases with advancing age and HBP diagnosis consequently becomes more frequent in individuals over the years, reaching close to 100% in individuals over 80 years of age^22^. Thus, the older the person is, the longer the they will have been affected by HBP, and therefore the lower the alcohol consumption. 

Given this information, it is necessary that nurses understand that the aging process is accompanied by changes in physical, psychological and social aspects of the individual, and that these changes often lead older adults to feeling powerless for depending on family members, generating hopelessness and depression, which makes them more vulnerable to consuming alcoholic beverages^23^. Thus, establishing a relationship of trust between the nursing professional and older adults is essential for detecting inadequate life habits, and with this the necessity of implementing actions to promote the health of this population.

This research found that the variables of education level, individual monthly income, monthly family income, physical activity and leisure activity had a significant association with the resilience variable. It is worth pointing out that individuals who had more years of education in their life have relied on the conditions and personal tools which have contributed to developing the meaning of life in their approach to resilience. Thus, external protective factors such as social support and appreciation that people receive from their environment can influence the formation of internal conditions, thus contributing to personal competence, problem-solving, and above all, autonomy^24^. 

It is possible to state that people with better financial conditions are favored in learning resilience compared to people of lower income due to their having greater access to resilience promoting factors, such as education level and social support^25^. 

In the holistic perspective of health, the psychological benefits of physical activity are as important as the physical benefits, since they can lessen and prevent emotional disturbances and somatic disorders. We can also add that people who perform physical activity achieve feelings of well-being, happiness, self-esteem and decreased stress and depression. In view of these benefits, it should be noted that performing physical exercise contributes to expanding social networks, stimulates the ability to share group emotions, develop dialogue, improve expressiveness, produce disinhibitions in order to contribute to a better state of mental, physical and emotional health that go beyond physical fitness^26^.

For older adults, the relationship between leisure and resilience is even stronger. Establishing a routine with distracting/entertaining activities contributes to older adults achieving better levels of well-being and quality of life. This can be configured as a resource that strengthens resilience, helping them cope with various risks associated with aging^27^. 

Thus, it is identified that health professionals, especially nurses working in *ESF* units, can create a conducive environment for older adults with HBP to perform leisure activities through activities carried out in the unit or in other places of the community where these individuals can periodically meet to carry out manual activities, dances, theater, poetry, recreation, exchanges in experience, and health education, among other leisure activities. Thus, they can contribute to improving self-esteem, well-being, life satisfaction and especially strengthening the resilience of older adults.

Among the reports of remarkable events in the lives of older adults with HBP in the present investigation, we can point out the loss (death) of loved ones, family conflicts and the diagnosis of the disease in themselves and/or in family members, thereby indicating that these are events with a negative connotation in the lives of these individuals. However, some authors clarify that negative experiences can make the person more resilient^28^.

Although no significant association between alcohol consumption/dependence and resilience was found in the study population, it is important to establish the relationship between these variables identified in the literature. 

Alcohol consumption can often be considered a coping strategy for stressful life events^29^, especially in people with changes in their mental health such as anxiety, depression and self-esteem^30^. In this context, resilience is an important feature to moderate the association between stress and negative emotions by providing the individual with the ability to adapt to stressful circumstances while maintaining their mental well-being^31^. It should be noted that few studies have been developed to directly verify the relationship between alcohol consumption/dependence and resilience, especially in the older adult population, but those found in the literature indicate an inverse relationship between these two variables, since resilience has been identified as a protective mechanism associated with a reduced risk of consuming alcoholic beverages^31^ ^-^ ^33^. 

In this context, it is perceived that stressful events are inevitable in life, however the “resilience” characteristic is key for explaining individual differences in psychological and behavioral improvement in confronting these events^31^. Thus, establishing a relationship between changes in self-esteem and negative events in life with alcohol consumption/dependence becomes clear, while resilience appears as a protective mechanism for these events that affect mental health and mitigate the impact of negative emotions, and thus reduce the consumption of alcoholic beverages^33^. This statement was not confirmed in the present study since a relationship between alcohol consumption/dependence and resilience in the older adults with HBP was not observed; a fact that can be explained by the different sociodemographic and economic characteristics of the study population, thus suggesting that new studies be conducted in different older adult populations.

It is worth mentioning that resilience can be improved through active learning. However, we point out that until now it has only been slightly stimulated in health services for preventing and treating alcohol consumption/dependence^34^.

As a limitation of the study we can point out the cross-sectional design of the research, which does not enable verifying the cause-effect relationship of the results found; however, it was possible to characterize and associate independent variables with dependent variables by observing the situation of the older adults with HBP at that moment. Another aspect that presented limitations refers to sampling, since a collection was not performed with all older adults with HBP; nevertheless, conducting a study with the total population would be difficult due to the high number of people with these characteristics. We emphasize that a sample calculation using a statistical program was adopted, thereby selecting a representative sample for this population.

## Conclusion

It has been found that most of the evaluated older adults are at low-risk for consuming alcoholic beverages. However, older adults who present risky consumption, harmful alcohol use and even a likely dependency for consuming alcohol have been identified. In addition, it was found that most of these people have moderately low to moderate resilience. It should be emphasized that a relevant percentage of those interviewed were classified with low resilience. No statistically significant association between alcohol consumption/dependence and resilience were identified in the present study.

We additionally emphasize that this study may bring contributions to the nursing area, since it presents the importance of nurses to stimulate resilience in older adults with HBP, as well as the need to include screening for alcohol consumption/dependence as a care routine in this population. Therefore, knowledge and understanding of this theme and an elaboration of actions for their promotion will favor quality of life, as well as the social and individual well-being for older adults with HBP.

Furtherore, we highlight the importance of further studies evaluating the relationship between resilience and alcohol consumption/dependence in older adults with HBP, considering that the consumption of alcoholic beverages concomitant to treatment of chronic diseases can be harmful. On the other hand, resilience can be considered as a fundamental part for these individuals to overcome obstacles and to better determine pharmacological and non-pharmacological treatment through changes in their life habits, thus strengthening their physical and mental health, and consequently improving their quality of life.
